# Substantial decline of organ preservation fluid contamination following adoption of ischemia-free liver transplantation: a post-hoc analysis

**DOI:** 10.1097/JS9.0000000000001163

**Published:** 2024-02-08

**Authors:** Jianwen Lin, Yefu Li, Tongdi Fang, Tielong Wang, Kang Liao, Qiang Zhao, Dongping Wang, Maogen Chen, Xiaofeng Zhu, Yinghua Chen, Honghui Chen, Yiwen Guo, Liqiang Zhan, Jiayi Zhang, Tao Zhang, Ping Zeng, Yaqin Peng, Lu Yang, Changjie Cai, Zhiyong Guo, Xiaoshun He

**Affiliations:** a Organ Transplant Center; Departments of b Private Medical Center; c Clinical Laboratory; d Anesthesiology; e Surgical Intensive Care Unit, The First Affiliated Hospital, Sun Yat-sen University; f Guangdong Provincial Key Laboratory of Organ Donation and Transplant Immunology; g Guangdong Provincial International Cooperation Base of Science and Technology (Organ Transplantation); h NHC key Laboratory of Assisted Circulation, Sun Yat-sen University, Guangzhou, China

**Keywords:** Ischaemia-free liver transplantation, preservation fluid contamination, recipient infection

## Abstract

**Introduction:**

Preservation fluid (PF) contaminations are common in conventional liver transplantation (CLT) and presumably originate from organ or PF exposures to the external environment in a non-strict sterile manner. Such exposures and PF contamination may be avoided in ischaemia-free liver transplantation (IFLT) because of the strict sterile surgical procedures. In this study, the authors evaluated the impact of IFLT on organ PF contamination.

**Methods:**

A post-hoc analysis using data from the first randomized controlled trial of IFLT was performed to compare the incidence, pathogenic spectrum of PF contamination, and incidence of early recipient infection between IFLT and CLT. Multivariable logistic regression was used to explore risk factors for PF contamination.

**Results:**

Of the 68 cases recruited in the trial, 64 were included in this post-hoc analysis. The incidence of culture-positive PF was 9.4% (3/32) in the IFLT group versus 78.1% (25/32) in the CLT group (*P*<0.001). Three microorganisms were isolated from PF in the IFLT group, while 43 were isolated in the CLT group. The recipient infection rate within postoperative day 14 was 3.1% (1/32) in the IFLT group vs 15.6% (5/32) in the CLT group, although this difference did not reach statistical significance (*P*=0.196). Multivariate analysis revealed that adopting IFLT is an independent protective factor for culture-positive PF.

**Conclusion:**

PF contamination is substantially decreased in IFLT, and IFLT application is an independent protective factor for PF contamination. Using rigorous sterile measures and effective antibiotic therapy during IFLT may decrease PF contamination.

## Introduction

Highlights

- Preservation fluid (PF) contaminations are common in liver transplantation (LT).
- This study aimed to evaluate the impact of ischaemia-free LT on PF contamination.
- PF contamination was substantially decreased in ischaemia-free LT.
- PF contamination decline may owe to strict sterility and effective antibiotics.

Early postoperative infections remain a significant cause of morbidity and mortality among transplant recipients^1^. Donor infections and organ or organ preservation fluid (PF) contamination from procurement to implantation are considered important sources of early post-transplant infections^2–4^. Intraoperative PF cultures usually assess infections derived from donor organs or PF contamination. Previous studies have investigated the clinical relevance of culture-positive PF. PF contamination rates in liver transplantation (LT) have been reported to be high in previous prospective studies, varying from 84.8 to 98.4%^5–7^, while the incidence of PF-related recipient infections is lower than 5%^4–8^. However, compared with recipients with culture-negative PF, recipients with culture-positive PF have higher infection and mortality rates^8,9^, greater graft loss^10–12^, increased acute rejection rate^13^, and more frequent graft dysfunction^6^.

Previous studies have focused on the precise microbiological examination of PF, the addition of antibiotics to PF, and preemptive antibiotic therapy for recipients with high-risk culture-positive PF to reduce the potential risk of PF contamination^4,6,7,9^. Although antibiotics can be used in PF during conventional LT (CLT) to treat contamination, their efficacy is limited as the organs are preserved under static, hypothermic, and hypoxic conditions. More importantly, rather than treating PF contamination, research on preventing PF contamination at the source is scarce. It has been generally perceived that PF contamination is mainly caused by superficial saprophytic flora^2,6,7,9^, indicating that most microorganisms recovered from PF presumably originate from exposing organs or PF to the external environment in a non-strict sterile manner. Such exposure is usually inevitable in CLT. Adopting a rigorous sterile technique from procurement to implantation may prevent PF contamination.

In 2017, we developed a novel LT procedure called ischaemia-free LT (IFLT)^14^. During IFLT, the donor’s liver is procured, preserved, and implanted under continuous normothermic machine perfusion (NMP). All the procedures are performed using a rigorous sterile technique and antibiotics is used under normothermic and oxygenated conditions. Thus, we hypothesized that IFLT could reduce PF contamination compared to CLT. Recently, a randomized controlled trial (RCT) assessing the safety and efficacy of IFLT versus CLT has been conducted in our centre^15,16^. In this study, we conducted a post-hoc analysis to compare the incidence, pathogenic spectrum, and clinical impact of PF contamination between IFLT and CLT.

## Methods

### Study design

**Figure 1:**
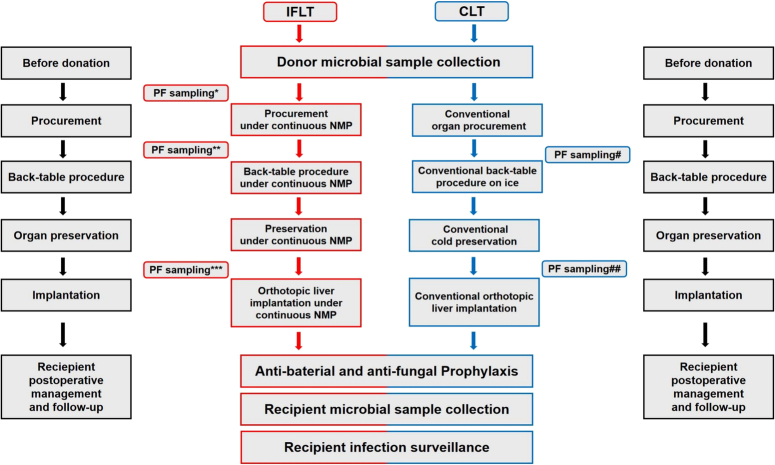
Trial design diagram regarding the specific surgical procedures, microbiological sampling time, recipient postoperative management, and follow-up. *, **, and ***: PF sampling before NMP, at the start of ex-situ NMP, and after NMP in IFLT, respectively; # and ##: PF sampling just before the back-table procedure and after cold preservation in CLT, respectively. CLT, conventional liver transplantation; IFLT, ischaemia-free liver transplantation; NMP, normothermic machine perfusion; PF, preservation fluid.

This investigator-initiated, open-label, phase III, prospective, single-centre RCT assessed the safety and efficacy of IFLT in patients with end-stage liver disease. Patients were screened and randomized in a ratio of 1:1 to receive either IFLT or CLT. The primary endpoint was the incidence of early allograft dysfunction. Details of the recruitment, randomization, sample size calculation, surgical procedures, follow-up, and study results are provided in previous reports^15,16^. Further details of the microbiological sampling time, recipient postoperative management, and follow-up are shown in Fig. 1.

The incidence and pathogenic spectrum of PF contamination, the microbiology of isolates from donor samples, and early recipient infection within 14 days postoperatively were compared between the IFLT and CLT groups. The risk factors for PF contamination were also analyzed in this post-hoc analysis. Other infection related indicators of recipients, including fever over 38.5°C, peak white blood cell (WBC) count, peak neneutrophil proportion (Neu%), peak C-reactive protein (CRP) levels and peak procalcitonin (PCT) levels within postoperative day 14, were also compared between the two groups. The study was compliant with the declaration of Helsinki and approved by The Research Ethics Committee of our hospital. All patients provided written informed consent to participate. The trial was registered on chictr.org.cn. The current study has been reported in line with the STROCSS criteria^17^, Supplemental Digital Content 1, http://links.lww.com/JS9/B848.

### Techniques of procurement, back-table procedure, and preservation

#### CLT procedure

After disinfecting the surgical area, an abdominal grand-cross incision was made. Cannulas were placed in the aorta, superior mesenteric vein, and inferior vena cava, and the liver was flushed *in situ* with a cold University of Wisconsin (UW) solution. The common bile duct was mobilized to the lowest possible level and transected. The gallbladder was incised, and the bile was flushed out. Crushed ice was placed in the abdominal cavity and around the liver for rapid cooling. The liver was retrieved with a diaphragm patch around the inferior vena cava and placed in 0–4°C UW solution. Gentamycin (240 000 units) was added to the UW solution, and a standard back-table preparation on ice was performed. The liver was then stored in ice before implantation.

#### IFLT procedure

**Figure 2:**
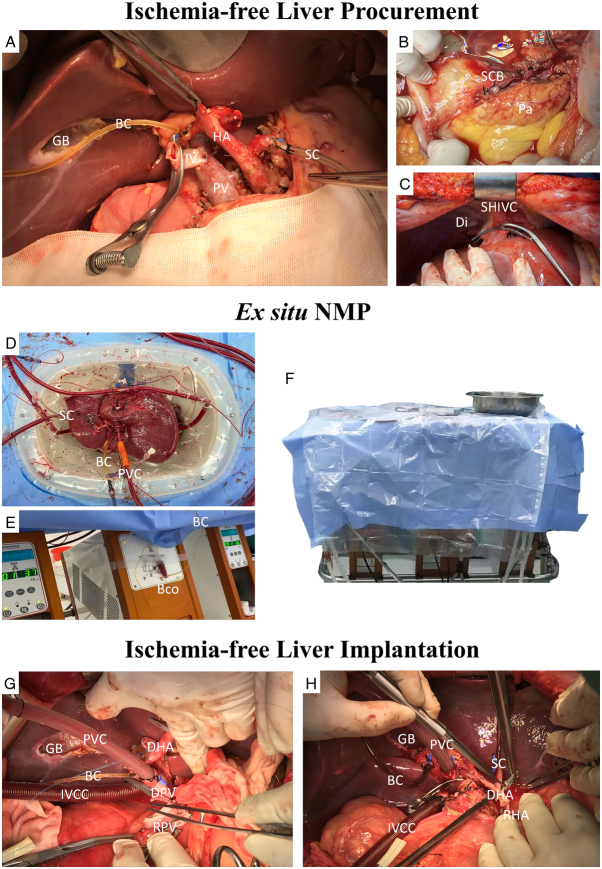
Ischemia-free liver transplant procedure. (A–C) Shows the sterile techniques during ischaemia-free liver procurement. The gallbladder was resected (A). The bile was drained well through a tube in the common bile duct (A). The common bile duct was transected at the upper margin of the pancreas without transection of the pancreas (B). Suprahepatic inferior vena cava was transected below the diaphragm (C). (D–F) Shows the sterile techniques during ex-situ normothermic machine perfusion (NMP). The NMP device was handled by well-trained personnel, using standard operating procedures and sterile disposables. The liver was perfused in an organ reservoir (D). The bile was drained continuously through the tube in the common bile duct to a bile container (D, E). When the perfusion was stable and no operation was required, the perfusion device was covered with a sterile sheet (F). (G, H) Shows the ischaemia-free liver implantation. The bile was drained continuously during vascular anastomosis (G, H), until biliary anastomosis. BC, bile duct cannula; Bco, bile container; Di, diaphragm; DHA, donor hepatic artery; DPV, donor portal vein; GB, gallbladder bed; HA, hepatic artery; IV, interposition vein for portal vein; IVCC, inferior vena cava cannula; Pa, pancreas; PV, portal vein; PVC, portal vein cannula in the interposition vein; RHA, recipient hepatic artery; RPV, recipient portal vein; SC, splenic artery cannula; SCB, stump of common bile duct; SHIVC, suprahepatic inferior vena cava.

The NMP device was primed with a red-blood-cell-based PF. Imipenem/cilastatin (1 g/1 g) and metronidazole (0.5 g) were added to the PF. After disinfecting the surgical area, an abdominal grand-cross incision was made. The liver was fully mobilized using precise techniques in the donor. Cannulas were inserted into the splenic or gastroduodenal artery, inferior vena cava, and portal vein and connected to the NMP device to establish an in-situ circuit. NMP was initiated, and the liver was harvested and transferred to the perfusion device under continuous NMP. The liver was then preserved on the perfusion device prior to implantation. Figure 2 shows the IFLT procedure.

Notably, ice was not required during IFLT. During donor liver procurement in IFLT, the gallbladder was resected under sterile conditions. Neither gallbladder nor biliary tract flushing was required. The bile was drained well before implantation through a tube placed in the common bile duct (Fig. 2A). Transection of the pancreas and diaphragm were not required (Fig. 2B and C). During ex-situ NMP, standard operating procedures and strict sterile measures were applied to ensure the sterility of the perfusion system. The NMP device was handled by well-trained perfusionists, using sterile disposables. The liver was perfused in an organ reservoir (Fig. 2D). The bile was drained continuously through the tube in the common bile duct to a bile container (Fig. 2D and E). PF samples were collected through ports on the perfusion line and infusions were performed through a port connected to the organ reservoir to minimize opening of organ reservoir. When the perfusion was stable and no operation was required, the perfusion device was covered with a sterile sheet (Fig. 2F). During implantation, the bile was drained continuously during vascular anastomosis (Fig. 2G and H), until biliary anastomosis. Comparisons of the technical characteristics before implantation between IFLT and CLT are shown in Table 1.

**Table 1:** Measures for preventing preservation fluid contamination during surgery before organ implantation in IFLT comparing to CLT.

	IFLT	CLT
Procurement		
Cooling the abdominal cavity with ice	No	Yes
Gallbladder resection during procurement	Yes	No
Gallbladder or biliary tract flushing	No	Yes
Transection of pancreas	No	Yes
Diaphragm opening	No	Yes
Back-table procedure		
Low temperature condition provided by ice	No	Yes
Organ preservation		
Bile external drainage	Yes	No
Preservation fluid		
Antibiotics in PF	Imipenem/cilastatin and metronidazole	Gentamicin

CLT, conventional liver transplantation; IFLT, ischaemia-free liver transplantation.

### Perioperative anti-microbial prophylaxis and immunosuppression protocols

Intravenous cefoperazone/sulbactam and caspofungin were used for perioperative prophylaxis. When the recipient was allergic to cefoperazone/sulbactam, moxifloxacin hydrochloride was used as an alternative prophylaxis antibiotics. The immunosuppression protocol included induction with Basiliximab and maintenance with Tacrolimus and Mycophenolate Mofetil. The anti-microbial prophylaxis and immunosuppression protocols were identical for both groups.

### Microbiological sample collection and processing

In the CTL group, the grafts were preserved in UW solution. PF samples from each case were obtained twice under sterile conditions (just before the back-table procedure and after cold preservation). In the IFLT group, the grafts were preserved under NMP. PF samples were obtained thrice (before NMP, at the start of ex-situ NMP, and after NMP). All PF samples were processed using the BacT/ALERT 3D system (bioMérieux, Marcy l’Etoile). The volume of PF aliquoted for culture was 10 ml. The inoculated bottles contained antibiotic neutralizers and were incubated for 6 days at 35°C before discharge. Microbial identification was performed using the VITEK 2 system (bioMérieux, Marcy l’Etoile). Antibiotic susceptibility tests for the strains were performed using Gram-negative susceptibility (GNS) cards on the VITEK system (bioMérieux, Marcy l’Etoile). Susceptibility test results were interpreted according to the criteria recommended by the Clinical and Laboratory Standards Institute^18^. *E. coli* ATCC 25922 and *K. pneumoniae* ATCC 700603 were used as quality control strains for susceptibility testing.

For both groups, cultures of blood, pleural and abdominal collections (if external drainage was present), sputum, bronchoalveolar lavage fluid, oral swabs, rectal swabs, and urine were obtained routinely at least once from the donors within 1 week before donation and from the recipients when they were in the ICU after LT. When donors or recipients were suspected of infection, more cultures were obtained at the corresponding site.

### Definition of PF contamination

PF contamination was defined as positive when one or more microorganisms were isolated from any PF sample, and negative when none were found. In the IFLT group, PF contamination before NMP was defined as positive culture from PF samples obtained before or at the start of ex-situ NMP; PF contamination during NMP was defined as positive culture from PF samples obtained after NMP without PF contamination before NMP. In the CLT group, PF contamination before the back-table procedure was defined as positive culture from PF samples obtained just before the back-table procedure; PF contamination during the back-table procedure or preservation was defined as positive culture from PF samples obtained after cold preservation without PF contamination before the back-table procedure or different microorganisms isolated from PF samples obtained after cold preservation.

Microorganisms cultured from PF were classified as “high-risk” and “low-risk” according to classification criteria proposed by previous studies. The following microorganisms were considered “high-risk”: *Staphylococcus aureus*, *β-hemolytic Streptococcus* species, *Streptococcus pneumoniae*, *Enterococcus* species, Gram-negative bacteria, spore-forming anaerobic Gram-positive bacteria, and fungi. All the others were considered “low-risk”^4,9^. Donor-related PF contamination was defined as contamination caused by the same microorganism isolated from any donor sample. Regarding counting the microorganism species in PF samples, identical microorganisms cultured at different times were counted only once.

Postoperative recipient infections were defined following the Centers for Disease Control and Prevention/National Healthcare Safety Network guidelines^19^. PF-related recipient infection was defined as a documented infection in the recipient by the same microorganism isolated from the PF culture^4^. One recipient infection event referred to an infection at one site in a patient that lasted for a certain period.

### Statistical analysis

We estimated the prevalence of culture-positive PF and early postoperative recipient infection and the microbiology of isolates obtained from donor and PF samples. All data are expressed as the means±standard deviation or percentages. For comparisons between groups, we used the χ^2^ test for categorical variables and the Student *t*-test and Mann–Whitney *U* test for continuous variables. A multivariate logistic regression analysis of factors potentially associated with culture-positive PF was performed, including all statistically significant variables in the univariate analysis, with odds ratios (OR) and 95% CI calculated. Data analyses were performed using SPSS 26.0 for Windows (SPSS). *P* less than 0.05 was considered statistically significant.

## Results

### Baseline characteristics

Of the 68 cases recruited in the trial, 64 were included in the post-hoc analysis, including 32 in the IFLT group and 32 in the CLT group. In the IFLT group, one transplant was cancelled because of fever before the operation, and informed consent was withdrawn in another case. In the CLT group, one transplant was cancelled because lung and bone metastases were discovered preoperatively by chance, while PF samples were lost in another case. The two groups were well-balanced regarding baseline demographic data, including age, sex, ICU stay length, and surgery time (Table 2). Notably, the microbiology of donor samples was comparable between the two groups. More details on the microorganisms isolated from the donors are shown in Supplementary Table 1, Supplemental Digital Content 2, http://links.lww.com/JS9/B849.

**Table 2:** Baseline characteristics of liver transplantation in the IFLT and CLT groups.

	IFLT (*n*=32)	CLT (*n*=32)
Donor		
Age, year	44.78±11.85	40.41±11.46
Sex, male, *n* (%)	22 (68.8)	21 (65.6)
Length of ICU stay, day	8.09±4.09	7.97±5.32
Donor with one or more positive cultures, *n* (%)	24 (75)	22 (68.8)
Organ external exposure time^a^, h	7.23±0.89	7.08±0.84
Recipient		
Age, year	54.09±9.48	51.34±11.02
Sex, male, *n* (%)	28 (87.5)	27 (84.3)
Length of ICU stay post-LT, day	1.92±1.39	3.01±2.91
Surgery-related data		
Length of recipient surgery, h	6.79±1.12	7.26±2.16

CLT, conventional liver transplantation; IFLT, ischaemia-free liver transplantation; LT, liver transplantation.

a Machine perfusion time in IFLT or cold ischaemia time in CLT.

### Prevalence of PF contamination

The prevalence of culture-positive PF was 9.4% (3/32) in the IFLT group and 78.1% (25/32) in the CLT group (OR 34.524, 95% CI 8.062–147.834, *P*<0.001) (Table 3), which has been reported in our previous study^16^. In the IFLT group, PF contamination occurred before NMP in two cases and during NMP in one case. No microorganism were isolated from the PF samples obtained at the start of ex-situ NMP (Supplementary Table 2, Supplemental Digital Content 3, http://links.lww.com/JS9/B850). In the CLT group, PF contamination occurred before the back-table procedure in 23 cases, and during the back-table procedure or preservation in 11 cases (Table 3).

**Table 3:** Comparisons of prevalence of preservation fluid contamination between the IFLT and CLT groups.

	IFLT (*n*=32), *n* (%)	CLT (*n*=32), *n* (%)	OR (95% CI)	*P*
PF contamination^a^	3 (9.4)	25 (78.1)	34.524 (8.062–147.834)	<0.001
PF contamination before NMP in IFLT^b^	2	NA		
PF contamination during NMP in IFLT^c^	1	NA		
PF contamination before back-table procedure in CLT^d^	NA	23		
PF contamination during back-table procedure or preservation in CLT^e^	NA	11		
PF contamination by “high-risk” microorganisms	1 (3.1)	15 (46.9)	27.353 (3.320–225.365)	<0.001
PF contamination by imipenem-resistant microorganisms	1 (3.1)	12 (37.5)	18.6 (2.241–154.345)	0.001
Donor-related PF contamination	0	8 (25)	NA	0.005

CLT, conventional liver transplantation; IFLT, ischaemia-free liver transplantation; NA, not assessed; NMP, normothermic machine perfusion; OR, odd ratio; PF, preservation fluid.

a PF with one or more positive cultures.

b Cultured positive from PF samples obtained before or at the start of ex-situ NMP in the IFLT group.

c Cultured negative from PF samples before and at the start of ex-situ NMP but positive from PF samples obtained after NMP in the IFLT group.

d Cultured positive from PF samples obtained just before back-table procedure in the CLT group.

e Cultured negative from PF samples obtained just before back-table procedure but positive from PF samples obtained after cold preservation, or cultured positive from PF samples obtained just before back-table procedure but different microorganism isolated from PF samples obtained after cold preservation in the CLT group.

In the CLT group, 46.9% (15/32) of the cases were positive for “high-risk” microorganisms in the PF, while only one case was “high-risk” microorganism positive in the IFLT group. Imipenem-resistant microorganisms were isolated from the PF in 37.5% (12/32) of cases in the CLT group and one case in the IFLT group (Table 3).

Donor-related PF contamination occurred in eight cases in the CLT group but none in the IFLT group (Table 3). Most (87.5%, 7/8) of these contaminations were caused by “high-risk” microorganisms. Further details on the microbiological characteristics of donor-related PF contamination are provided in Supplementary Table 3, Supplemental Digital Content 4, http://links.lww.com/JS9/B851.

### Microbiology of PF samples in IFLT and CLT

Three microorganisms were isolated from the PF in the IFLT group and 43 in the CLT group. In the CLT group, 46.5% (20/43) of the PF isolates were classified as “high-risk” microorganisms, most were Gram-negative bacteria (*n*=11), and seven fungi were isolated. The microorganisms isolated from the PF are summarized in Table 4.

**Table 4:** Microbiology of preservation fluid samples in the IFLT and CLT groups.

	IFLT	CLT
Microorganisms isolated from PF	3	43
Microorganisms isolated before NMP in IFLT^a^	2	NA
Microorganisms isolated during NMP in IFLT^b^	1	NA
Microorganisms isolated before the back-table procedure in CLT^c^	NA	30
Microorganisms isolated during the back-table procedure or preservation in CLT^d^	NA	13
“High-risk” microorganism	1 (1/3, 33.3%)	20 (20/43, 46.5%)
Gram-positive bacteria	1	2
* Enterococcus faecium*	1	1
* Staphylococcus aureus*	0	1
Gram-negative bacteria	0	11
* Klebsiella pneumoniae*	0	5
* Stenotrophomonas maltophilia*	0	2
* Pseudomonas aeruginosa*	0	1
* Proteus mirabilis*	0	1
* Citrobacter fraudi*	0	1
* Corynebacterium afermentans*	0	1
Fungi	0	7
* Candida albicans*	0	2
* Candida tropicalis*	0	2
* Candida glabrata*	0	1
* Candida krroux*	0	1
* Trichosporon asahii*	0	1
“Low-risk” microorganism	2 (2/3, 66.7%)	23 (23/43, 53.5%)
* Staphylococcus epidermidis*	0	10
* Staphylococcus hominis*	1	3
* Staphylococcus haemolyticus*	0	3
* Staphylococcus warneri*	0	3
* Staphylococcus cohnii*	0	1
* Lactobacillus acidophilus*	0	1
* Propionibacterium acnes*	0	1
* *Other Gram-positive bacilli	1	1
Anaerobic bacterium	0	0
Microorganisms resistant to imipenem	1 (1/3, 33.3%)	18 (18/43, 41.9%)

CLT, conventional liver transplantation; IFLT, ischaemia-free liver transplantation; NA, not assessed; NMP, normothermic machine perfusion; PF, preservation fluid.

a Microorganisms isolated from PF samples obtained before NMP in the IFLT group.

b Microorganisms isolated from PF samples obtained after NMP in the IFLT group.

c Microorganisms isolated from PF samples obtained just before the back-table procedure in the CLT group.

d Microorganisms isolated from PF samples obtained after cold preservation in the CLT group, excluding identical microorganisms that were isolated from PF samples obtained just before the back-table procedure.

In the CLT group, 41.9% (18/43) of the PF isolates were resistant to imipenem (Table 4), including seven fungi and six methicillin-resistant CNS (MRSCON) (Supplementary Table 4, Supplemental Digital Content 5, http://links.lww.com/JS9/B852). One MRSCON was identified in the IFLT group (Supplementary Table 4, Supplemental Digital Content 5, http://links.lww.com/JS9/B852).

### Risk factors associated with culture-positive PF

Possible risk factors, such as donor age, sex, length of ICU stay, donors with positive culture, donor organ external exposure time, cause of death, surgical procedures adopted (IFLT or CLT), operational transfusion, and length of recipient surgery time, were included in the analysis. Among these, the adoption of IFLT (OR 0.029, 95% CI 0.007–0.124, *P*<0.001) and donor age (OR 0.948, 95% CI 0.904–0.993, *P*=0.024) were found to be risk factors in the univariate analysis. In the multivariate analysis, only IFLT was an independent protective factor for culture-positive PF (OR 0.029, 95% CI 0.007–0.124, *P*<0.001). The results of the risk factor analysis are summarized in Table 5.

**Table 5:** Univariable and multivariable analyses of factors for culture-positive preservation fluid in liver transplantation.

	Univariable logistic regression		Multivariable logistic regression	
Variables	OR (95% CI)	*P*	OR (95% CI)	*P*
Surgery-related data				
* *Adopting IFLT	0.029 (0.007–0.124)	<0.001	0.029 (0.007–0.124)	<0.001
* *Organ external exposure time^a^	0.995 (0.985–1.005)	0.340		
* *Operational transfusion of RBC	1.000 (1.000–1.000)	0.484		
* *Length of recipient surgery	1.000 (0.999–1.001)	0.793		
Donor				
* *Age	0.948 (0.904–0.993)	0.024	0.941 (0.881–1.006)	0.073
* *Sex	1.682 (0.587–4.818)	0.333		
* *Length of ICU stay	0.974 (0.874–1.086)	0.633		
* *Donor with positive culture	0.514 (0.171–1.548)	0.237		
Cause of death				
* *Trauma	0.723 (0.266–1.969)	0.526		
* *CVA	1.413 (0.487–4.100)	0.525		
* *Hypoxia	0.758 (0.141–4.075)	0.746		
* *Other	0.000 (0.000–0.000)	1.000		

CVA, cerebrovascular accident; IFLT, ischaemia-free liver transplantation; OR, odd ratio; RBC, red blood cell.

a Machine perfusion time in IFLT or cold ischaemia time in CLT.

### Recipient infection within 14 days postoperatively

The recipient infection rate on postoperative day 14 was 3.1% (1/32) in the IFLT group and 15.6% (5/32) in the CLT group; however, the difference was not statistically significant (*P*=0.196). One and six infection events were recorded in the IFLT and CLT groups, respectively. The lungs were the most common infection sites (*n*=1 in the IFLT group and *n*=4 in the CLT group). Infection with positive culture was observed in three cases in the CLT group and none in the IFLT group. No PF-related recipient infections were observed in any group. Incidence of fever over 38.5°C, peak WBC, peak Neu%, and peak CRP level within postoperative day 14, were comparable between the two groups. Notably, the peak PCT level was higher in the CLT versus IFLT group, although the difference was not statistically significant (*P*=0.091) (Table 6).

**Table 6:** Comparisons of recipient infection within POD 14 between the IFLT and CLT groups.

	IFLT (*n*=32)	CLT (*n*=32)	OR (95% CI)	*P*
Recipient infections within POD 14	1 (3.1%)	5 (15.6%)	5.741 (0.631–52.234)	0.196
Infection events	1	6		
Infection site				0.120
* *Pulmonary	1	4		
* *Urinary tract	0	1		
* *Central venous catheter-related	0	1		
* *Infection with positive culture	0	3		NA
Culture site				NA
* *Pulmonary	0	1		
* *Urinary tract	0	1		
* *Central venous catheter-related	0	1		
* *PF-related recipient infection	0	0		NA
Fever >38.5°C within POD 14	11 (34.4%)	11 (34.4%)	1 (0.356**–**2.806)	1.000
Peak WBC within POD 14, mean (SD), 10^9^/l	10.16 (5.03)	10.56 (3.50)		0.716
Peak Neu% within POD 14, mean (SD)	0.90 (0.04)	0.89 (0.04)		0.256
Peak CRP within POD 14, mean (SD), mg/l	133.37 (68.72)	155.88 (56.58)		0.162
Peak PCT within POD 14, mean (SD), ug/l	14.08 (24.95)	29.77 (45.09)		0.091
Postoperative hospital stay, median (IQR), day	18 (16–24)	17 (14–26)		0.631

CLT, conventional liver transplantation; CRP, C-reactive protein; IFLT, ischaemia-free liver transplantation; IQR, interquartile range; NA, not assessed; Neu%, neutrophil proportion; OR, odd ratio; PCT, procalcitonin; PF, preservation fluid; POD, postoperative day; WBC, peripheral white blood cell counting.

## Discussion

The incidence, microbiology, and clinical impacts of PF contamination were compared for the first time between CLT and the newly developed IFLT in this post-hoc analysis of our previous RCT. Overall, we found that PF contamination declined substantially to a remarkably low level in IFLT; in contrast, it was as high as previously reported in CLT^5–7^. In addition, we found that applying IFLT was an independent protective factor against PF contamination based on the risk factor analysis in our study.

Because the main source of PF contamination is probably exposure of the donor organ or PF to the external environment during procedures before implantation, avoiding such exposure may be ideal to reduce PF contamination. Since the blood supply to the donor organ can be maintained throughout the process of IFLT, surgeons do not need to operate as hurriedly as during rapid organ procurement in CLT. This allows them to pay more attention to keeping the surgical field sterile. Because ice is not required during IFLT, contamination by melted ice and cavity viscera injury by the sharp edges of the ice can be avoided. Owing to the novel design of bile drainage in IFLT, the surgical field and donor organs under preservation are not exposed to bile. In addition, contamination from transection of the pancreas, or even injury to the digestive tract or urinary tract, can also be avoided owing to sufficient time for a precise operation. Moreover, contamination from the thoracic cavity can be prevented because the diaphragm does not need to be cut open. Therefore, rigorous sterility during IFLT may contribute to a much lower PF contamination rate.

Potent antibiotics in the PF may be another advantage of asepsis in IFLT. Several studies have demonstrated that either hypothermic machine perfusion (MP) or NMP plus antibiotics can effectively eliminate bacteria inside the tissue of the target organ after 3 h^20–22^. However, data on the effects of excluding microorganisms in the PF are unavailable. On the other hand, it has recently been reported that NMP may increase the microorganism population in PF and cause severe sepsis in recipients^23^. In this case report, PF in NMP was contaminated with *E. coli* resistant to antibiotics used in PF and for postoperative prophylaxis, and the recipient experienced severe sepsis caused by the same pathogen after LT^23^. Another study demonstrated that the growth of *Staphylococcus aureus* and *Staphylococcus epidermidis* in PF was enhanced by warmer and nutrient-rich conditions during NMP^24^. These results suggest that NMP can potentially create a beneficial environment for microbial growth and may cause severe infections. In our study, we isolated only one microorganism from the PF after NMP, and no subsequent recipient infection was observed, suggesting the powerful effects of broad spectrum antibiotics (imipenem) during ex-situ NMP in the IFLT group.

To determine the major reason for the much lower PF contamination rate in the IFLT group, we analyzed the prevalence of PF contamination at different time points. It has been reported that the incidence of PF contamination was as high as 42–56% before MP for conventionally retrieved organs^25,26^. In contrast, no microorganism was isolated from PF samples obtained at the start of ex-situ NMP in the IFLT group. Moreover, PF contamination occurred before the back-table procedure in 71.9% (23/32) of the cases in the CLT group, accounting for 92% (23/25) of the cases with PF contamination. At this time point, the antibiotics in the PF did not have enough time to take effects on microorganisms. Importantly, even if imipenem were used in the PF of the CLT group, the PF contamination rate would still be high because 41.9% (18/43) of the isolates were imipenem-resistant in the CLT group. Therefore, it is reasonable to deduce that the lower PF contamination rate in the IFLT group should be mainly explained by stricter sterile measures during donor liver procurement (to prevent entry of microorganisms to PF), instead of by more powerful antibiotic administration (to inhibit growth of microorganisms in PF).

As previously reported, only ~13–28% of the isolates from PF were considered “high-risk” pathogens in CLT^4–8^; in contrast, it was as high as 46.9% in our study. Further analysis revealed that the median length of ICU stay of donors in our trial was approximately 8 days (Table 2), while it was ~2–4 days in previous studies^4–7^. Many studies have demonstrated that prolonged ICU stay is associated with a higher risk of infection or colonization of virulent pathogens, including *Klebsiella pneumoniae* and *Candida* spp^27–36^, coincidently, the main “high-risk” isolates from PF in our trial. Thus, prolonged ICU stays can increase the risk of infection or colonization by “high-risk” pathogens in donors, and the PF might be exposed to these pathogens during procurement. In addition, we identified donor-related PF contamination in eight cases; seven were caused by “high-risk” microorganisms, accounting for 46.7% (7/15) of PF positive for “high-risk” microorganisms. Collectively, the prolonged ICU stay of donors may explain the higher incidence of PF positive for “high-risk” pathogens in our series. Notably, the length of ICU stay of donors in the IFLT group was comparable to that in the CLT group and longer than that previously reported in CLT, suggesting the importance of strict sterile techniques in preventing PF contamination.

The current study demonstrated the effectiveness of IFLT in reducing PF contamination. However, our study had some limitations. First, this was a single-centre trial with a small sample size. A multicenter trial with a larger sample size is now ongoing to confirm the results of the current study and to explore whether IFLT can reduce recipient infection rate. Second, the type and pharmacokinetics of the antibiotics differed between the two groups. The exact contribution of strict sterile measures and potent antibiotics in the reduction of PF contamination remains unclear. In addition, metagenomic examination was not used to detect microorganisms; the incidence of PF contamination and recipient infections might be underestimated. Finally, due to the limitation of commercially available NMP devices in China, IFLT can be done in only a few centres. With the development of a commercially available device for IFLT, we believe the IFLT technique can be practiced on a wider scale. These limitations provide a scope for future research.

Overall, this study is the first to report a post-hoc data analysis from an RCT in which PF contamination can be substantially reduced with IFLT. The use of rigorous sterile measures and effective antibiotic therapy during IFLT may have contributed to remarkably low levels of PF contamination.

## Ethical approval

The study was compliant with the declaration of Helsinki and approved by The Research Ethics Committee of the first affiliated hospital of Sun Yat-sen university. Reference number: [2019]037.

## Consent

All patients provided written informed consent to participate.

## Source of funding

This study was supported by grants as follows: the National Natural Science Foundation of China (82170663, 82070670, and 81970564), the Guangdong Provincial Key Laboratory Construction Projection on Organ Donation and Transplant Immunology (2013A061401007, 2017B030314018), Guangdong Provincial International Cooperation Base of Science and Technology (Organ Transplantation) (2015B050501002), Science and Technology Program of Guangzhou (201704020150), Science and Technology Program of Guangdong (2020B1111140003), Sun Yat-sen University Young Teacher Key Cultivate Project (17ykzd29), and “Elite program” specially supported by China Organ Transplantation Development Foundation.

## Author contribution

X.H., Z.G., and J.L. had full access to all of the data in the study and take responsibility for the integrity of the data and the accuracy of the data analysis. All authors had access to the study results and reviewed and approved the final version of the manuscript for publication. X.H. served as the principal investigator for the study, contributed to study design, interpreted data, and drafted the manuscript; Z.G. contributed to study design, interpreted data, and drafted the manuscript; J.L., T.F., Y.L., and T.W. collected, analyzed and interpreted data, and drafted the manuscript; K.L. and Y.P. contributed to microbiological samples processing; Q.Z., D.W., M.C., X.Z., and Y.C. conducted the operations and postoperative follow-ups; H.C., Y.G., L.Z., J.Z., and T.Z. served as perfusionists and collected the perfusion data and perfusate samples; P.Z. contributed to collect the clinical data; L.Y. conducted surgical anaesthesia and contributed to surgery-related data collection; C.C. contributed to donor management and donor microbiological sample collection.

## Conflicts of interest disclosure

The authors declare no conflicts of interest.

## Research registration unique identifying number (UIN)

Clinical trial registration: chictr.org. ChiCTR1900021158.

## Guarantor

Xiaoshun He (gdtrc@163.com), Zhiyong Guo (rockyucsf1981@126.com).

## Data availability statement

Data analyzed during the current study are available upon reasonable request.

## Provenance and peer review

Not commissioned, externally peer-reviewed.

## Supplementary Material
