# Supplementary material for: Substantial decline of organ preservation fluid contamination following adoption of ischemia-free liver transplantation: a post-hoc analysis
Source: Int J Surg. 2024 Feb 8;110(5):2855–64. doi: 10.1097/JS9.0000000000001163 (PMC11093427; doi:10.1097/JS9.0000000000001163)
Supplement: Supplementary file 2 [file js9-110-2855-s002.docx]

**Supplementary Table 1.** **Comparisons of the Microbiology of Donor Samples Between the IFLT and CLT groups**

|  | **IFLT** | **CLT** | ***P*** |
| --- | --- | --- | --- |
| **Microorganisms isolated** | **63** | **71** | **0.311** |
| **Gram-positive cocci** | **6** | **13** |  |
| *Staphylococcus aureus* | 2 | 8 |  |
| *Staphylococcus epidermidis* | 2 | 3 |  |
| *Staphylococcus haemolyticus* | 0 | 1 |  |
| *Enterococcus faecium* | 1 | 1 |  |
| *Enterococcus avis* (group D) | 1 | 0 |  |
| **Gram-negative bacilli** | **27** | **30** |  |
| *Pseudomonas aeruginosa* | 8 | 13 |  |
| *Acinetobacter baumannii* | 10 | 2 |  |
| *Klebsiella pneumoniae* | 3 | 7 |  |
| *Burkholderia cepacia* | 2 | 1 |  |
| *Haemophilus influenzae* | 1 | 1 |  |
| *Escherichia coli* | 2 | 0 |  |
| *Enterobacter aerogenes* | 0 | 2 |  |
| *Enterobacter hormaechei* | 0 | 1 |  |
| *Burkholderia multivorans* | 0 | 1 |  |
| *Pseudomonas fluorescens* | 1 | 0 |  |
| *Prevotella intermedia* | 0 | 1 |  |
| *Stenotrophomonas maltophilia* | 0 | 1 |  |
| **Fungi** | **30** | **28** |  |
| *Candida albicans* | 9 | 16 |  |
| *Candida glabrata* | 13 | 4 |  |
| *Candida parapsilosis* | 4 | 0 |  |
| *Candida tropicalis* | 2 | 2 |  |
| *Candida palmitata* | 1 | 0 |  |
| *Candida nivaliana* | 0 | 1 |  |
| *Candida famata* | 0 | 1 |  |
| *Paecilomyces variotii* | 0 | 2 |  |
| *Kodamaea ohmeri* | 1 | 1 |  |
| *Aspergillus niger* | 0 | 1 |  |
| **Specimen** | **63** | **71** | **0.407** |
| Blood | 5 | 4 |  |
| Central venous catheter | 1 | 0 |  |
| Bronchoalveolar lavage fluid | 10 | 15 |  |
| Sputum | 16 | 24 |  |
| Oral swab | 21 | 18 |  |
| Urine | 2 | 4 |  |
| Urinary catheter | 1 | 0 |  |
| Stool | 3 | 0 |  |
| Rectal swab | 4 | 6 |  |
| **“High-risk” microorganism** | **62** | **67** | **0.370** |
| **Microorganisms resistant to imipenem** | **40** | **38** | **0.293** |

IFLT, ischemia-free liver transplantation; CLT, conventional liver transplantation.
