# Supplementary material for: Substantial decline of organ preservation fluid contamination following adoption of ischemia-free liver transplantation: a post-hoc analysis
Source: Int J Surg. 2024 Feb 8;110(5):2855–64. doi: 10.1097/JS9.0000000000001163 (PMC11093427; doi:10.1097/JS9.0000000000001163)
Supplement: Supplementary file 3 [file js9-110-2855-s003.docx]

**Supplementary Table 2.** **Cases of Preservation Fluid Contamination in the IFLT group**

| Case number | Microorganisms isolated before NMP | Microorganisms isolated at the start of *ex situ* NMP | Microorganisms isolated after NMP | NMP duration,  hour | Imipenem-  resistant  microorganism | Recipient infection |
| --- | --- | --- | --- | --- | --- | --- |
| IFLT-03 | *Enterococcus faecium* | None | None | 6.85 | No | No |
| IFLT-10 | None | None | *Staphylococcus hominis* | 8.2 | Yes | No |
| IFLT-21 | Gram-positive bacillus | None | None | 7 | No | No |

IFLT, ischemia-free liver transplantation; NMP, normothermic machine perfusion.
