# Supplementary material for: Substantial decline of organ preservation fluid contamination following adoption of ischemia-free liver transplantation: a post-hoc analysis
Source: Int J Surg. 2024 Feb 8;110(5):2855–64. doi: 10.1097/JS9.0000000000001163 (PMC11093427; doi:10.1097/JS9.0000000000001163)
Supplement: Supplementary file 4 [file js9-110-2855-s004.docx]

**Supplementary Table 3.** **Cases of Donor-Related Preservation Fluid Contamination in the CLT group**

| Case number | Microorganisms involved | Donor samples | Donor-related  PF contamination with “high-risk” microorganism |
| --- | --- | --- | --- |
| CLT-06 | *Staphylococcus aureus,*  *Pseudomonas aeruginosa* | Sputum | Yes |
| CLT-07 | *Klebsiella pneumoniae* | Sputum | Yes |
| CLT-12 | *Staphylococcus epidermidis* | Blood | No |
| CLT-13 | *Klebsiella pneumoniae* | Bronchoalveolar lavage fluid | Yes |
| CLT-15 | *Klebsiella pneumoniae* | Bronchoalveolar lavage fluid | Yes |
| CLT-21 | *Candida albicans* | Oral swab  Rectal swab | Yes |
| CLT-26 | *Candida tropicalis* | Rectal swab | Yes |
| CLT-28 | *Candida albicans* | Oral swab | Yes |

CLT, conventional liver transplantatio; PF, preservation fluid.
