# Supplementary material for: Substantial decline of organ preservation fluid contamination following adoption of ischemia-free liver transplantation: a post-hoc analysis
Source: Int J Surg. 2024 Feb 8;110(5):2855–64. doi: 10.1097/JS9.0000000000001163 (PMC11093427; doi:10.1097/JS9.0000000000001163)
Supplement: Supplementary file 5 [file js9-110-2855-s005.docx]

**Supplementary Table 4.** **Cases of Preservation Fluid Contamination by Imipenem-Resistant Microorganisms in the IFLT and CLT groups**

| Case number | Microorganisms involved | Sampling time |
| --- | --- | --- |
| IFLT-10 | *Staphylococcus hominis* | After NMP |
| CLT-02 | *Staphylococcus hominis* | Just before the back-table procedure |
|  | *Candida glabrata* | After cold preservation |
| CLT-04 | *Staphylococcus epidermidis* | Just before the back-table procedure |
|  |  | After cold preservation |
| CLT-06 | *Staphylococcus aureus* | Just before the back-table procedure |
|  | *Pseudomonas aeruginosa* | Just before the back-table procedure |
|  |  | After cold preservation |
| CLT-09 | *Stenotrophomonas maltophilia*  *Staphylococcus haemolyticus*  *Candida tropicalis* | Just before the back-table procedure |
|  |  | After cold preservation |
| CLT-11 | *Staphylococcus cohnii* | After cold preservation |
| CLT-12 | *Candida albicans* | Just before the back-table procedure |
|  |  | After cold preservation |
| CLT-13 | *Candida tropicalis* | After cold preservation |
| CLT-15 | *Candida albicans* | After cold preservation |
| CLT-19 | *Staphylococcus epidermidis* | After cold preservation |
|  | *Candida krroux* | After cold preservation |
| CLT-21 | *Staphylococcus epidermidis* | After cold preservation |
| CLT-22 | *Stenotrophomonas maltophilia*  *Trichosporon asahii* | Just before the back-table procedure |
| CLT-27 | *Lactobacillus acidophilus* | Just before the back-table procedure |
|  |  | After cold preservation |

IFLT, ischemia-free liver transplantation; CLT, conventional liver transplantation; NMP, normothermic machine perfusion.
